# Development and Preliminary Validation of the SHUDE Scoring System for Chronic Endometritis in Women With Previous Reproductive Failure Undergoing In Vitro Fertilization Treatment

**DOI:** 10.7759/cureus.116711

**Published:** 2026-09-22

**Authors:** Shuchi Jain, Deepti Shrivastava, Sunita Vagha

**Affiliations:** 1 Obstetrics and Gynecology, Mahatma Gandhi Institute of Medical Sciences, Warud, IND; 2 Obstetrics and Gynecology, Jawaharlal Nehru Medical College, Datta Meghe Institute of Medical Sciences (Deemed to Be University), Wardha, IND; 3 Pathology, Jawaharlal Nehru Medical College, Datta Meghe Institute of Medical Sciences (Deemed to Be University), Wardha, IND

**Keywords:** chronic endometritis, content validity, cronbach's alpha, hysteroscopy, infertility, ivf, recurrent implantation failure, reproductive failure, scoring system, shude score

## Abstract

Background

Chronic endometritis (CE) has been increasingly recognized in women with infertility and previous reproductive failure, but its diagnosis remains challenging because clinical, hysteroscopic, and histopathological findings may vary between individuals. Hysteroscopy provides direct visualization of the endometrial cavity; however, individual hysteroscopic findings may have limited diagnostic value when considered in isolation. The present study aimed to develop and pilot-test the SHUDE scoring system, a standardized multidomain scoring framework for the structured assessment of CE in women with previous reproductive failure undergoing in vitro fertilization.

Methods

Following approval from the Institutional Ethics Committee, this methodological study was conducted at a tertiary care center. Thirty eligible women with infertility and a history of reproductive failure were included for the pilot assessment of the scoring system. The SHUDE scoring system comprised six domains: demographic characteristics, reproductive history, clinical history, clinical examination, hysteroscopic findings, and evidence of endometrial tuberculosis. Content validity was assessed using a four-point relevance scale by a multidisciplinary panel of six experts comprising reproductive medicine specialists, obstetricians and gynecologists, and clinicians experienced in diagnostic hysteroscopy. The item-level content validity index (I-CVI) and scale-level content validity index based on the average method (S-CVI/Ave) were calculated. Internal consistency was assessed using Cronbach's alpha.

Results

The six domains demonstrated acceptable content validity, with I-CVI values ranging from 0.83 to 1.00 and an overall S-CVI/Ave of 0.94. Pilot testing among 30 eligible women yielded a Cronbach's alpha of 0.84, indicating good internal consistency across the six domains.

Conclusion

The SHUDE scoring system demonstrated high content validity and good internal consistency in this preliminary evaluation. These findings provide initial support for the relevance and coherence of the scoring framework but do not establish its diagnostic accuracy or an optimal diagnostic threshold. Further evaluation in larger, independent populations is required to determine its diagnostic performance, reproducibility, and potential clinical applicability.

## Introduction

Chronic endometritis (CE) is a persistent inflammatory condition of the endometrium that has been increasingly recognized among women with infertility and previous reproductive failure [1]. Although CE may be clinically asymptomatic or associated with nonspecific symptoms, it has been reported to be associated with adverse reproductive outcomes, including miscarriage and implantation failure. Studies among women undergoing assisted reproductive technologies have demonstrated an association between CE and poorer reproductive outcomes, while treatment of appropriately diagnosed CE has been associated with improved reproductive outcomes in selected populations [2,3].

The diagnosis of CE remains challenging because no single diagnostic criterion has been universally accepted. Histological identification of endometrial stromal plasma cells is commonly used for diagnosis, and immunohistochemical staining for syndecan-1 (CD138) facilitates the detection of these cells [4]. However, the distribution of plasma cells within the endometrium may be heterogeneous, and differences in diagnostic thresholds and interpretation can contribute to variability in the diagnosis of CE [5].

Hysteroscopy provides direct visualization of the endometrial cavity and is therefore used as a supportive method for identifying features that may be associated with CE. Reported hysteroscopic findings include diffuse or focal endometrial hyperemia, hemorrhagic spots, abnormal vascular patterns, micropolyps, and endometrial polyps [6,7]. However, the diagnostic relevance of individual hysteroscopic findings may vary, and their interpretation can be influenced by observer experience. Standardized hysteroscopic criteria have consequently been proposed to improve the consistency of assessment of findings associated with CE [8].

Given the limitations of relying on individual hysteroscopic features, combining multiple clinically relevant parameters into a structured scoring system may provide a more systematic approach to assessment. Liu et al. developed a hysteroscopic scoring system for CE that incorporates several morphological findings and demonstrated the potential value of combining them rather than evaluating them individually [9]. Subsequent studies have continued to evaluate hysteroscopic features and structured assessment approaches while highlighting variability in diagnostic performance across different criteria and study populations [5].

The development of a clinical scoring system requires systematic evaluation of the relevance of its individual components. Content validity is an important initial step in instrument development and can be quantitatively assessed using the content validity index (CVI) based on expert evaluation [10]. When multiple clinical parameters are combined in a scoring system, assessing internal consistency can provide preliminary information about the coherence of the included components. Cronbach's alpha is commonly used for this purpose [11].

Considering the limitations associated with assessment based on individual hysteroscopic findings, the present study aimed to develop the SHUDE scoring system as a standardized multidomain approach for the structured assessment of CE in women with previous reproductive failure undergoing in vitro fertilization (IVF) treatment. The objective was to develop the scoring system using clinically relevant domains and to evaluate its preliminary content validity and internal consistency through expert assessment and pilot testing.

## Materials and methods

Study design and setting

This methodological study was conducted in the Department of Obstetrics and Gynecology, with support from the Department of Pathology, at Jawaharlal Nehru Medical College, Sawangi (M), Wardha. The study received approval from the Institutional Ethics Committee of Datta Meghe Institute of Medical Sciences, Sawangi, Wardha (IEC reference number DMIMS (DU)/IEC/2022/844) and was registered with the Clinical Trials Registry-India (CTRI) under registration number CTRI/2025/08/092834. Written informed consent was obtained from all participants after the study procedures were explained.

Study Population

Women of reproductive age with a history of reproductive failure undergoing infertility evaluation were consecutively recruited for the pilot assessment of the SHUDE scoring system. Reproductive failure was defined according to the study criteria as a history of failed embryo implantation following previous IVF treatment and/or previous pregnancy loss. Women were eligible if they had normal hysterosalpingography findings, normal karyotyping, negative screening for antiphospholipid syndrome, and normal thyroid and prolactin levels.

Women were excluded if they had unexplained uterine bleeding during the preceding month, had received antibiotics within one month before endometrial biopsy, had uterine malformations, had a submucosal myoma or endometrial polyp greater than 5 mm, or reported a history of smoking or alcohol use.

Sample Size

For the development and preliminary validation of the SHUDE scoring system, a multidisciplinary expert panel comprising six subject-matter experts was constituted to assess content validity. Following expert review and refinement of the scoring system, 30 eligible women were included in the pilot sample for preliminary assessment of internal consistency. This sample represented the pilot phase of instrument development and was not intended to provide definitive estimates of diagnostic accuracy.

Development of the SHUDE scoring system

The SHUDE scoring system was developed through a review of the relevant literature and consultation with clinicians experienced in reproductive medicine, obstetrics and gynecology, and diagnostic hysteroscopy. The scoring system comprised six domains: demographic characteristics, reproductive history, clinical history, clinical examination, hysteroscopic findings, and evidence of endometrial tuberculosis.

The demographic domain included age and duration of married life, while the reproductive history domain included previous implantation failure or pregnancy loss. The clinical history domain comprised chronic pelvic pain, previous treatment for acute or chronic pelvic inflammatory disease, vaginal discharge, abnormal uterine bleeding, and a history of tubo-ovarian mass or pelvic surgery.

The clinical examination domain comprised predefined findings recorded during routine gynecological examination, including suprapubic tenderness on per-abdominal examination; unhealthy vaginal discharge and cervical congestion on per-speculum examination; and restricted uterine mobility, uterine tenderness, and fornicial fullness on per-vaginal examination.

The hysteroscopic domain included diffuse endometrial hyperemia, focal hyperemia, hemorrhagic spots, dilated endometrial vessels, micropolyps, and endometrial polyps. The sixth domain recorded evidence suggestive of endometrial tuberculosis.

The individual parameters were assigned predefined scores based on the available literature and expert clinical assessment during the development process.

Content Validation

The preliminary SHUDE scoring system was reviewed by six clinicians representing reproductive medicine, obstetrics and gynecology, and diagnostic hysteroscopy. The panel comprised two reproductive medicine specialists, two obstetricians and gynecologists, and two clinicians experienced in diagnostic hysteroscopy. For the purpose of this study, clinicians experienced in diagnostic hysteroscopy were those with clinical experience in performing and interpreting diagnostic hysteroscopic examinations in women undergoing infertility evaluation.

Each expert independently assessed the relevance, clarity, and clinical applicability of the proposed items using a four-point relevance scale ranging from 1 (not relevant) to 4 (highly relevant). Feedback from the expert panel was considered during refinement of the scoring system.

Content validity was assessed using the item-level content validity index (I-CVI) and the scale-level content validity index based on the average method (S-CVI/Ave). For each item, the I-CVI was calculated as the proportion of experts assigning a rating of 3 or 4. The S-CVI/Ave was calculated as the mean of the I-CVI values across the items. An I-CVI of ≥0.78 and an S-CVI/Ave of ≥0.80 were considered acceptable [10].

Pilot Testing and Reliability

The finalized SHUDE scoring system was pilot-tested among 30 women who fulfilled the predefined eligibility criteria. Internal consistency was assessed using Cronbach's alpha. Corrected item-total correlations and the change in Cronbach's alpha after deletion of individual items were also examined to assess the contribution of individual components to the overall scoring system [11]. A Cronbach's alpha of ≥0.70 was considered acceptable for internal consistency.

Hysteroscopic assessment and endometrial sampling

Hysteroscopy was performed three to five days after menstruation using a 30° hysteroscope with a 3.5 mm outer sheath and 0.9% saline as the distension medium. The endometrial cavity was systematically examined, and predefined hysteroscopic findings were recorded. Hysteroscopic images were independently interpreted by two clinicians experienced in diagnostic hysteroscopy.

Following hysteroscopy, an endometrial sample was obtained using a Pipelle sampler. The specimen was fixed and processed for immunohistochemical staining with CD138 (syndecan-1) to identify endometrial stromal plasma cells. Appropriate positive and internal controls were used for immunohistochemical assessment. All specimens were evaluated by the same pathologist, who was blinded to the hysteroscopic findings. According to the study protocol, the presence of more than five CD138-positive plasma cells in the endometrial stroma was considered indicative of CE.

Statistical analysis

Statistical analysis was performed using IBM SPSS Statistics version 29.0 (IBM Corp., Armonk, NY, USA). Categorical variables were summarized as frequencies and percentages, while continuous variables were summarized using appropriate descriptive statistics. Content validity was assessed using the I-CVI and S-CVI/Ave. Internal consistency was assessed using Cronbach's alpha, corrected item-total correlations, and Cronbach's alpha if an item was deleted. An I-CVI of ≥0.78, an S-CVI/Ave of ≥0.80, and a Cronbach's alpha of ≥0.70 were considered acceptable according to the predefined study criteria [10,11].

## Results

A multidisciplinary panel of six experts participated in the content validation of the SHUDE scoring system. The panel comprised two reproductive medicine specialists, two obstetricians and gynecologists, and two clinicians experienced in diagnostic hysteroscopy, providing representation from the principal clinical disciplines involved in the evaluation of CE and reproductive failure (Table 1).

**Table 1:** Characteristics of the expert panel (n=6)

Characteristic	Frequency
Reproductive medicine specialists	2
Obstetricians and gynecologists	2
Clinicians experienced in diagnostic hysteroscopy	2
Total experts	6

All six domains of the preliminary SHUDE scoring system demonstrated acceptable content validity. The I-CVI values ranged from 0.83 to 1.00, with complete agreement among experts for four of the six domains. The clinical history and evidence of endometrial tuberculosis domains had I-CVI values of 0.83. The overall S-CVI/Ave was 0.94, indicating excellent overall content validity of the scoring system (Table 2).

**Table 2:** Content validity of the SHUDE scoring system I-CVI, item-level content validity index; S-CVI/Ave, scale-level content validity index based on the average method Ratings of 3 or 4 on the four-point relevance scale were considered indicative of item relevance.

Domain	Experts rating as relevant (3 or 4), n	I-CVI
Demographic characteristics	6	1.00
Reproductive history	6	1.00
Clinical history	5	0.83
Clinical examination	6	1.00
Hysteroscopic findings	6	1.00
Evidence of endometrial tuberculosis	5	0.83
Overall S-CVI/Ave	-	0.94

The finalized six-domain SHUDE scoring system was pilot-tested in 30 eligible women. The overall Cronbach's alpha was 0.84, demonstrating good internal consistency of the scoring system and supporting the retention of the six domains for further clinical evaluation (Table 3).

**Table 3:** Internal consistency reliability of the SHUDE scoring system

Parameter	Value
Pilot sample size	30
Number of domains	6
Cronbach's alpha	0.84

The overall preliminary validation findings demonstrated high content validity and good internal consistency of the SHUDE scoring system. The six domains were retained following expert assessment, with I-CVI values ranging from 0.83 to 1.00 and an S-CVI/Ave of 0.94. The pilot reliability assessment yielded a Cronbach's alpha of 0.84 (Table 4).

**Table 4:** Summary of preliminary validation of the SHUDE scoring system I-CVI, item-level content validity index; S-CVI/Ave, scale-level content validity index based on the average method I-CVI and S-CVI/Ave were calculated using the average method.

Analysis	Result	Interpretation
Number of experts	6	Multidisciplinary expert panel
Number of domains	6	Six-domain scoring system
I-CVI	0.83-1.00	Acceptable to excellent item-level content validity
S-CVI/Ave	0.94	Excellent overall content validity
Pilot sample	30	Preliminary reliability assessment
Cronbach's alpha	0.84	Good internal consistency

## Discussion

The present study describes the development and preliminary validation of the SHUDE scoring system for the structured assessment of CE in women with previous reproductive failure undergoing IVF treatment. The scoring system demonstrated high content validity, with I-CVI values ranging from 0.83 to 1.00 and an overall S-CVI/Ave of 0.94. Pilot assessment also demonstrated good internal consistency, with a Cronbach’s alpha of 0.84. These findings indicate that the domains included in the SHUDE scoring system were considered relevant and clinically applicable by the expert panel and demonstrated satisfactory coherence when considered within the scoring framework.

CE has attracted considerable interest in reproductive medicine because of its reported association with infertility, recurrent implantation failure, and recurrent pregnancy loss [1-3]. The condition may be clinically subtle, and its diagnosis remains challenging because studies have used heterogeneous histological criteria, sampling approaches, and diagnostic methods [4]. CD138 immunohistochemistry has become an important method for identifying endometrial stromal plasma cells; however, variation in plasma-cell distribution and tissue sampling may influence diagnostic interpretation [5,12,13]. These considerations highlight the potential value of integrating clinical and hysteroscopic information with histopathological assessment rather than relying on a single feature or diagnostic approach.

Hysteroscopy provides direct visualization of the endometrial cavity and has been extensively investigated as an adjunctive method for the assessment of CE. Findings reported in association with CE include diffuse or focal hyperemia, hemorrhagic spots, abnormal vascular patterns, micropolyps, and endometrial polyps [6]. However, individual hysteroscopic findings may have variable diagnostic performance, and their interpretation can be influenced by observer experience. Consequently, structured approaches that combine relevant hysteroscopic findings may help improve the consistency of clinical assessment [14].

The concept of combining hysteroscopic findings into a composite score is not new. Liu et al. developed a hysteroscopic scoring system for CE and reported that a combination of hysteroscopic findings could provide useful diagnostic information [9]. Subsequent studies have continued to investigate structured hysteroscopic assessment while demonstrating variability in the diagnostic performance of individual findings and scoring approaches [14,15]. The SHUDE scoring system extends this concept through a multidomain approach. In addition to hysteroscopic findings, it incorporates demographic characteristics, reproductive history, clinical history, clinical examination findings, and evidence of endometrial tuberculosis. The intention is to provide a structured framework in which multiple clinically relevant features can be considered together rather than relying on an isolated hysteroscopic observation.

An important finding of the present study was the high content validity of the SHUDE scoring system. Four of the six domains received complete agreement among the experts, while the remaining two domains had an I-CVI of 0.83. The overall S-CVI/Ave of 0.94 indicates a high level of agreement regarding the relevance of the included domains. Content validity is an important component of clinical instrument development because it evaluates whether the components of an instrument adequately represent the construct that the instrument is intended to assess [10]. The inclusion of experts from reproductive medicine, obstetrics and gynecology, and clinicians experienced in diagnostic hysteroscopy provided input from different clinical perspectives relevant to reproductive failure and CE. However, the expert panel did not include a pathologist; therefore, pathology-specific content expertise was not directly represented during the formal content-validation process. Histopathological assessment was performed separately as part of the clinical evaluation, and inclusion of pathology expertise in future refinement of the instrument may further strengthen its multidisciplinary development.

The reliability assessment provided additional preliminary evidence regarding the SHUDE scoring system. The Cronbach’s alpha of 0.84 indicated good internal consistency across the six domains. This suggests that the components demonstrated satisfactory coherence within the overall scoring framework. Nevertheless, Cronbach’s alpha should be interpreted specifically as a measure of internal consistency and not as evidence of diagnostic validity. A satisfactory alpha value does not establish the ability of the SHUDE score to distinguish women with CE from those without the condition, nor does it establish an appropriate diagnostic cutoff [11]. Such properties require evaluation against an appropriate reference standard in a larger diagnostic-validation study.

Accordingly, the findings of the present study should be regarded as preliminary evidence supporting the content validity and internal consistency of the SHUDE scoring system rather than as evidence of established diagnostic accuracy or clinical efficacy. The present findings provide a basis for subsequent evaluation of the instrument in independent populations. Future studies should assess diagnostic discrimination, determine an appropriate cutoff value, and evaluate diagnostic accuracy against CD138 immunohistochemistry and other relevant reference assessments. Inter-observer reliability should also be examined, particularly for the clinical examination and hysteroscopic components, because these assessments may be influenced by examiner experience.

Strengths and limitations

A major strength of the study was the structured development of the SHUDE scoring system using literature-informed domains and multidisciplinary expert assessment, followed by pilot testing of internal consistency. However, the small expert panel and single-center pilot sample of 30 participants limit the breadth and generalizability of the findings. Although participants were recruited consecutively using predefined eligibility criteria, selection and spectrum bias cannot be completely excluded because the study population represented a specific infertility and previous reproductive failure population. Finally, diagnostic accuracy, the optimal diagnostic threshold, and inter-observer reliability were not assessed. These parameters require evaluation in larger, independent cohorts before broader clinical application of the SHUDE scoring system.

## Conclusions

The present study demonstrated that the SHUDE scoring system incorporates clinically relevant domains for the structured assessment of CE in women with previous reproductive failure undergoing IVF treatment. The scoring system showed high content validity and good internal consistency in the preliminary assessment. However, these findings do not establish its diagnostic accuracy or define an optimal diagnostic threshold. Further evaluation in larger, independent populations is required to assess its diagnostic performance, reproducibility, and potential clinical applicability.
